# Mapping tuberculosis treatment outcomes in Ethiopia

**DOI:** 10.1186/s12879-019-4099-8

**Published:** 2019-05-28

**Authors:** Kefyalew Addis Alene, Kerri Viney, Darren J. Gray, Emma S. McBryde, Maereg Wagnew, Archie C. A. Clements

**Affiliations:** 1 Research School of Population Health, College of Health and Medicine, The Australian National University, Canberra, ACT Australia; 2 Institute of Public Health, College of Medicine and Health Sciences, University of Gondar, Gondar, Ethiopia; 3 Department of Public Health Sciences, Karolinska Institutet, Stockholm, Sweden; 4 Australian Institute of Tropical Health and Medicine, James Cook University, Townsville, Queensland Australia; 5 Ethiopia Federal Ministry of Health, Addis Ababa, Ethiopia; 6 Faculty of Health Sciences, Curtin University, Perth, Western Australia Australia

**Keywords:** Mapping, Spatial patterns, Tuberculosis, Treatment outcomes

## Abstract

**Background:**

Tuberculosis (TB) is the leading cause of death from an infectious disease in Ethiopia, killing more than 30 thousand people every year. This study aimed to determine whether the rates of poor TB treatment outcome varied geographically across Ethiopia at district and zone levels and whether such variability was associated with socioeconomic, behavioural, health care access, or climatic conditions.

**Methods:**

A geospatial analysis was conducted using national TB data reported to the health management information system (HMIS), for the period 2015–2017. The prevalence of poor TB treatment outcomes was calculated by dividing the sum of treatment failure, death and loss to follow-up by the total number of TB patients. Binomial logistic regression models were computed and a spatial analysis was performed using a Bayesian framework. Estimates of parameters were generated using Markov chain Monte Carlo (MCMC) simulation. Geographic clustering was assessed using the Getis-Ord Gi* statistic, and global and local Moran’s I statistics.

**Results:**

A total of 223,244 TB patients were reported from 722 districts in Ethiopia during the study period. Of these, 63,556 (28.5%) were cured, 139,633 (62.4%) completed treatment, 6716 (3.0%) died, 1459 (0.7%) had treatment failure, and 12,200 (5.5%) were lost to follow-up. The overall prevalence of a poor TB treatment outcome was 9.0% (range, 1–58%). Hot-spots and clustering of poor TB treatment outcomes were detected in districts near the international borders in Afar, Gambelia, and Somali regions and cold spots were detected in Oromia and Amhara regions. Spatial clustering of poor TB treatment outcomes was positively associated with the proportion of the population with low wealth index (OR: 1.01; 95%CI: 1.0, 1.01), the proportion of the population with poor knowledge about TB (OR: 1.02; 95%CI: 1.01, 1.03), and higher annual mean temperature per degree Celsius (OR: 1.15; 95% CI: 1.08, 1.21).

**Conclusions:**

This study showed significant spatial variation in poor TB treatment outcomes in Ethiopia that was related to underlying socioeconomic status, knowledge about TB, and climatic conditions. Clinical and public health interventions should be targeted in hot spot areas to reduce poor TB treatment outcomes and to achieve the national End-TB Strategy targets.

**Electronic supplementary material:**

The online version of this article (10.1186/s12879-019-4099-8) contains supplementary material, which is available to authorized users.

## Background

Tuberculosis (TB), a bacterial disease caused by *Mycobacterium tuberculosis*, and is the leading cause of death from an infectious disease worldwide [1]. TB kills more than one million people every year [1, 2]. Most deaths from TB could be prevented with early diagnosis and appropriate treatment. Despite enormous improvement in TB treatment outcomes that have led to the prevention of 53 million deaths between 2000 and 2016, there are still major problems with the treatment of TB [2]. Poor TB treatment outcomes are disproportionately high in low-income countries and among low-socio-economic groups within countries. Identifying patterns of TB treatment outcome across geographic areas is particularly relevant for TB control programs and health care providers for planning, implementing, monitoring, and evaluating control and prevention efforts to those areas at highest risk.

The Global Burden of Disease (GBD) study [1, 3] and the World Health Organization (WHO) [2] have produced national-level estimates of morbidity and mortality from TB on an annual basis. However, TB treatment outcome for administrative units of countries such as districts may differ significantly from the national average. Sub-national level analyses are important for planning purposes and to determine where services can further be enhanced.

Spatial epidemiological studies have assessed the incidence rates of TB in some parts of Ethiopia [4–9]. However, to date, no spatial epidemiological analyses of TB treatment outcomes have been reported in Ethiopia. There have been studies conducted in Iran and Argentina that reported variability in TB treatment outcomes across districts with low socio-economic status and difficult access to public transportation [10, 11]. Understanding the spatial distribution of TB treatment outcomes is important for measuring the effect of investments in TB control, and for measuring achievements of End TB strategies at the local level.

We integrated the national TB surveillance data and the Ethiopian Demographic and Health Survey (EDHS) data to quantify geographic variation of TB treatment outcomes in Ethiopia at the district level and to identify socio-economic, behavioural, health care access, and climatic factors associated with spatial clustering of poor TB treatment outcomes.

## Methods

### Study area

Ethiopia is located in East Africa and shares boundaries with Eritrea to the North, Djibouti and Somalia to the east, Sudan and South Sudan to the West, and Kenya to the South. It is the second-most populous country in Africa. The last official census report, which was undertaken in 2007, estimated a total population of 74 million [12]. Subsequent projections raised the population estimate to 102 million people in 2017 [13]. Approximately 85% of the population live in rural areas [12]. With a total area of about 1.1 million square kilometres, Ethiopia is the 10th and 27th largest country in Africa and in the world, respectively. The country has a variety of geographical features; its altitude ranges from 125 m below sea level at the Danakil Depression to 4620 m above sea level at mount Ras Dashen, it contains the source of the Blue Nile and is bisected by the Great Rift Valley.

Ethiopia is administratively divided into nine regional states and two city administrative councils (Addis Ababa and Dire Dawa). Each regional state is further divided into zones, districts (“woreda”), and neighbourhoods (“kebeles”). The districts are the decentralized administrative level and kebeles, within districts, are the lowest administrative unit in Ethiopia.

TB is the leading infectious cause of death in Ethiopia [14], killing more than 30 thousand people annually [15, 16]. Ethiopia is one of 30 high TB and MDR-TB burden countries [17]. The national population based TB prevalence survey conducted in 2010–2011 revealed that the prevalence of smear-positive TB among adults and all age group was found to be 108 and 63 per 100,000 head of population, respectively [18]. According to WHO’s 2014 Global TB report, Ethiopia has achieved all the three targets of the Millennium Development Goals (MDG) for TB prevention and control [19]. Mortality and prevalence due to TB has declined by more than 50% and the incidence rate is falling significantly [19]. However, in the last few years, the TB treatment success rate has decreased in Ethiopia (from 91% in 2012 to 84% in 2016) [2, 20, 21].

### Data sources

#### Tuberculosis data

District-level TB data (reported from June 2015 to June 2017) were obtained from the Health Management Information System (HMIS) managed by the national TB and leprosy control program (NTLCP). The data include the number of TB patients enrolled in directly observed therapy, short-course (DOTS) centres in each district with their treatment outcomes (including the number of patients with the TB treatment outcomes of cured, treatment completed, died, treatment failed, and lost to follow-up).

TB treatment outcomes were defined according to NTLCP guideline definitions [22], which have been adopted from WHO TB treatment guidelines [23]. In this study, a poor treatment outcome was defined as the sum of failure, death and lost to follow up, and treatment success was defined as the sum of cured and treatment completed. The study included pulmonary and extra-pulmonary drug-susceptible TB. Table 1 shows the definition of TB treatment outcomes.

**Table 1:** definitions of treatment outcomes for tuberculosis patients

Treatment Outcomes	Definitions
Cured	A pulmonary TB patient with bacteriologically confirmed TB at the beginning of treatment who was smear- or culture-negative in the last month of treatment and on at least one previous occasion.
Treatment completed	A TB patient who completed treatment without evidence of failure but with no record to show that sputum smear or culture results in the last month of treatment and on at least one previous occasion were negative, either because tests were not done or because results are unavailable.
Treatment failure	A TB patient whose sputum smear or culture is positive at month 5 or later during treatment.
Death	A TB patient who dies for any reason during the course of treatment.
Lost to follow-up	A TB patient whose treatment was interrupted for two consecutive months or more.
Poor treatment outcome	The sum of failure, death and lost to follow up.
Treatment success	The sum of cured and treatment completed.

### Independent variables

Data sources for the independent variables are provided in Table 2. Data on the knowledge and attitudes of the population regarding TB were obtained from the EDHS 2011 [24]. Data on health care access and data on behavioural factors such as chat chewing and alcohol drinking were obtained from the 2016 EDHS [25]. Socio-economic data such as population density, dependency ratio, average number of people in the household, unemployed rate, and literacy rate were obtained from the Ethiopia Atlas of Population Density, and the wealth index was obtained from the 2016 EDHS [25]. Climatic and environmental data such as Enhanced Vegetation Index, rainfall, aridity, and mean temperature were obtained from the EDHS Spatial Analysis repository [26].

**Table 2:** Summary of independent variables, sources of data and definition of variables

Independent variables	Data sources	Definition
Socio-economic factors		
Low wealth index	EDHS 2016	Total number of people with low wealth index (poorer and poorest) divided by the total number of people participated in the survey.
Population density	Ethiopia Atlas of Population Density	Number of people per square kilometre
Dependency ratio	Ethiopia Atlas of Population Density	Number of children (aged under 15 years) and elderly (aged 65+) dividing by the working-age population (aged 15–64 years)
Average number of persons per room	Ethiopia Atlas of Population Density	Average number of people living in a room
Unemployed population	Ethiopia Atlas of Population Density	Percentage of people in the labour force who were unemployed
Adult literacy rate	Ethiopia Atlas of Population Density	Percentage of population aged 15 years and above who had attended higher than secondary school or who can read and write a short simple statement
Behavioural factors		
Chat chewing	EDHS 2016	Total number of people chewing chat in the last 1 month prior to the survey divided by the total number of people participating in the survey
Alcohol drinking	EDHS 2016	Total number of people drinking alcohol in the month prior to the survey divided by the total number of people participating in the survey
Health care access and knowledge and attitude regarding TB		
Health care access problem	EDHS 2016	Difficulty of getting advice or treatment due to lack of money, or distance to a health facility
Good knowledge toward TB	EDHS 2011	Number of people with good knowledge towards TB divided by the total number of people participating in the survey.
Good attitude towards TB	EDHS 2011	Number of people with good attitude towards TB divided by the total number of people participating in the survey
Climatic and environmental factors		
Enhanced vegetation index	EDHS Spatial Analysis data	The average enhanced vegetation index which is calculated by measuring the density of green leaves in the near-infrared and visible bands.
Rainfall	EDHS Spatial Analysis data	Annual mean rainfall (mm)
Aridity	EDHS Spatial Analysis data	The average aridity index calculated by dividing the actual evapotranspiration by the potential evapotranspiration.
Mean temperature	EDHS Spatial Analysis data	Annual mean environmental air temperature (°C).

### Knowledge and attitudes regarding TB

Data on the knowledge and attitudes of the population regarding TB were obtained from the EDHS 2011 survey (as the EDHS 2016 survey did not include these data) [24, 25]. These data were collected by semi-structured questions by means of an interviewer-administered questionnaire. The TB knowledge of a person was assessed by three questions: 1) “have you ever heard of an illness called tuberculosis or TB (yes/no)”; 2) “how can a person get tuberculosis or TB?” and 3) “what symptoms will a person with tuberculosis or TB have?” A person was categorised as having “good” knowledge about TB if the person had ever heard about TB, correctly mentioned the route of transmission (i.e. TB is transmitted through the air when coughing or sneezing or through drinking of unboiled milk), and if the person mentioned at least one TB symptom (i.e. persistent cough for more than 2 weeks, weight loss, poor appetite, night sweats, chest pain or fever). Those who missed one or more of these three items were categorized as having ‘poor’ knowledge about TB. The attitude of a person towards TB was measured by two questions: 1) can tuberculosis or TB be cured (yes, no, or don’t know) and 2) if a member of your family got tuberculosis or TB, would you want it to remain a secret? A “good” attitude was defined by a person believing that TB can be cured and not wanting a family member’s TB to be kept a secret. A “poor” attitude was defined by a person believing that TB cannot be cured or wanting a family member’s TB diagnosis to be kept a secret.

#### Health care access

The health care access data were obtained from the 2016 EDHS [25]. As maternal and newborn health are priorities for the Government of Ethiopia [27], in the 2016 EDHS, the health care access data were collected only from women. Women (aged 15–49 years) were asked whether payment for advice or treatment, or the distance to a health facility, presented major problems in seeking medical advice or treatment for themselves when they were sick. We considered that healthcare access issues existed if either of these challenges were identified by the participants. A total of 15,683 women responded to these questions, of whom 9479 (60%) reported having at least one of the specified problems in accessing health care.

#### Behavioural factors

Data on behavioural factors such as chat chewing and alcohol drinking were obtained from the 2016 EDHS [25]. Chat chewing was assessed in the 2016 EHDS by asking the questions: 1) “have you ever chewed chat (yes/no)”; and 2) “during the last 30 days how many days did you chew chat?” Similarly, alcohol drinking was assessed by questions such as: 1) “have you ever taken a drink that contains alcohol (Tella/Tegi/Areke/Beer/Wine, etc...) (yes/ no)”; 2) “during the last 30 days, how many days did you have a drink that contains alcohol?” and 3) “during the last 13 months, how often did you take a drink that contains alcohol (almost every day, at least once a week, less than once a week, none in the last 13 months)?”. Since the smoking of cigarettes was rare among the 2016 EDHS participants (less than 1% of women and 4% of men smoke any type of tobacco), we did not include cigarette smoking in our study as an independent variable.

#### Socio-economic factors

Socio-economic data such population density, dependency ratio (defined as number of children (aged under 15 years) and elderly (aged 65+) divided by the working-age population (aged 15–64 years)), average number of people in the household, unemployed rate, and literacy rate were obtained from the Ethiopia Atlas of Population Density, and the wealth index was obtained from the 2016 EDHS [25].

The wealth index was calculated in the 2016 EDHS at the household level**.** Households were given scores based on the number and kinds of assets they own, ranging from a television to a bicycle or car, in addition to housing characteristics such as source of drinking water, toilet facilities, and flooring materials. These scores were derived using principal component analysis. National wealth quintiles were compiled by assigning the household score to each usual (de jure) household member, ranking each person in the household population by her or his score, and then dividing the distribution into five equal categories, each comprising 20% of the population. We considered those in the poorest and poorer wealth index quintiles as having a low wealth index and those in the middle, richer, and richest wealth quintiles as having a high wealth index. We then calculated the percentage of patients with a low wealth index in each zone as the total number of people with a low wealth index divided by the total number of people who had information on assets in the zone.

#### Climatic and environmental factors

Climatic and environmental data such as Enhanced Vegetation Index, rainfall, aridity, and mean temperature were obtained from the EDHS Spatial Analysis repository [26]. The EDHS program georeferenced these climatic and environmental data with the existing demographic and health survey data to use for spatial analysis.

#### Non-spatial statistical analyses

The prevalence of poor TB treatment outcomes was calculated by dividing the sum of death, treatment failure and lost to follow up by the total number of TB patients enrolled in the DOTS program. First, a univariate analysis was performed by taking the prevalence of poor TB treatment outcomes as the dependant variable and the geographical covariates as independent variables. Since the prevalence of poor TB treatment outcomes at zonal level was skewed to the right, the log transformed prevalence of poor TB treatment outcome was used in the univariate analysis. Variables with a *p*-value less than 0.2 in the univariate analysis were selected for the final model and checked for the presence of multi-collinearity using variance inflation factors (VIF), excluding variables with a VIF > 7. When we checked for the presence of multi-collinearity, a high degree of collinearity was observed among variables from within the same group of independent variables. Thus, we selected one variable from each group for the final model to avoid the observed multi-collinearity. The variables for the final model were selected first by eliminating implausible variables and then by using a p-value. The variable that was found to be more statistically significant was selected for the final spatial models. Low wealth index, chat chewing, poor knowledge towards TB, and annual mean temperature were selected for the final spatial models.

#### Spatial autocorrelation analysis

Spatial clustering of poor TB treatment outcomes was explored at a global scale using Moran’s I statistic and at a local scale using Local indicators of spatial association (LISA), estimated using the Anselin Local Moran’s I statistic, and the Getis-Ord statistic. The global Moran’s I statistic was used to assess the presence, strength and direction of spatial autocorrelation over the whole study area and to test the assumption of spatial independence in the implementation of the spatial pattern analysis. The LISA and the Getis-Ord statistics were used to detect local clustering of poor TB treatment outcomes and to identify the locations of hot-spots. These analyses were conducted using tools provided in ArcGIS.

#### Bayesian spatial analysis

Four different Bayesian models were constructed: unstructured model (Model I), spatially structured model without covariates (Model II), spatially structured model with covariates (Model III), and spatially structured and unstructured model with covariates (Model IV). Model IV includes all the components in the preceding models. These models were constructed using WinBUGS version 1.4.3 software (Medical Research Council Biostatistics Unit, Cambridge, United Kingdom). The details of the models are presented in the Additional file 1: Table S1.

We assumed that the observed prevalence of poor TB treatment outcome (r) at zone (*i*) had a binomial distribution with a total number of TB patients enrolled for treatment at the zone (*n*_*i*_) and the predicted prevalence of poor treatment outcomes at the zone (*p*_*i*_): *r*_*i*_ *~ Binomial (n*_*i*_*, p*_*i*_*).* Model IV for *p*_*i*_ was specified as follows:

$$ logit\ \left({p}_i\right)=\alpha +\sum \limits_N{\beta}_n\ast {\mathrm{X}}_{n,i}+{U}_i+{\mathrm{V}}_i $$

Where *p*_*i*_ is the probability of a poor treatment outcome in zone *i*; *α* is the intercept; $\sum \limits_N{\beta}_n\ast {\mathrm{X}}_{n,i}$ is the matrix of independent zone-specific variables (X, i.e. proportion of the population with a low wealth index, chat chewing and poor knowledge towards TB, and mean annual temperature) measured at each zone *i*, multiplied by their coefficients (*β*); *U*_*i*_ are unstructured random effects; *V*_*i*_ are the spatially structured random effects, modelled using conditional autoregressive (CAR) approach. The CAR component was defined using an adjacency matrix to determine the spatial relationships between zones. The adjacency matrix for each zone was generated using ArcGIS based on the queen definition, whereby two areas are considered neighbours if they share a common boundary or vertex. A weight of 1 was given if two zones were neighbours and a weight of 0 was given if two zones were not neighbours.

The posterior parameters were estimated using a Bayesian Markov Chain Monte Carlo (MCMC) simulation. Non-informative priors were specified for the intercept *α* (a non-informative, improper prior with bounds - ∞ and + ∞) and the coefficients (normal prior with mean = 0 and precision 1× 10^− 6^). The priors for the precision of the unstructured and spatially structured random effects were assigned non-informative gamma distributions with shape and scale parameters set at 0.001. The deviance information criterion (DIC) statistic was calculated to select the best-fitting model (models with a lower DIC statistic are considered to show a better compromise between model fit and parsimony). The model was run for 1,000,000 iterations and convergence was successfully achieved after 900,000 iterations. Convergence of the models was determined by visual inspection of posterior kernel density and history plots.

## Results

### Tuberculosis treatment outcomes in Ethiopia

**Fig. 1:**
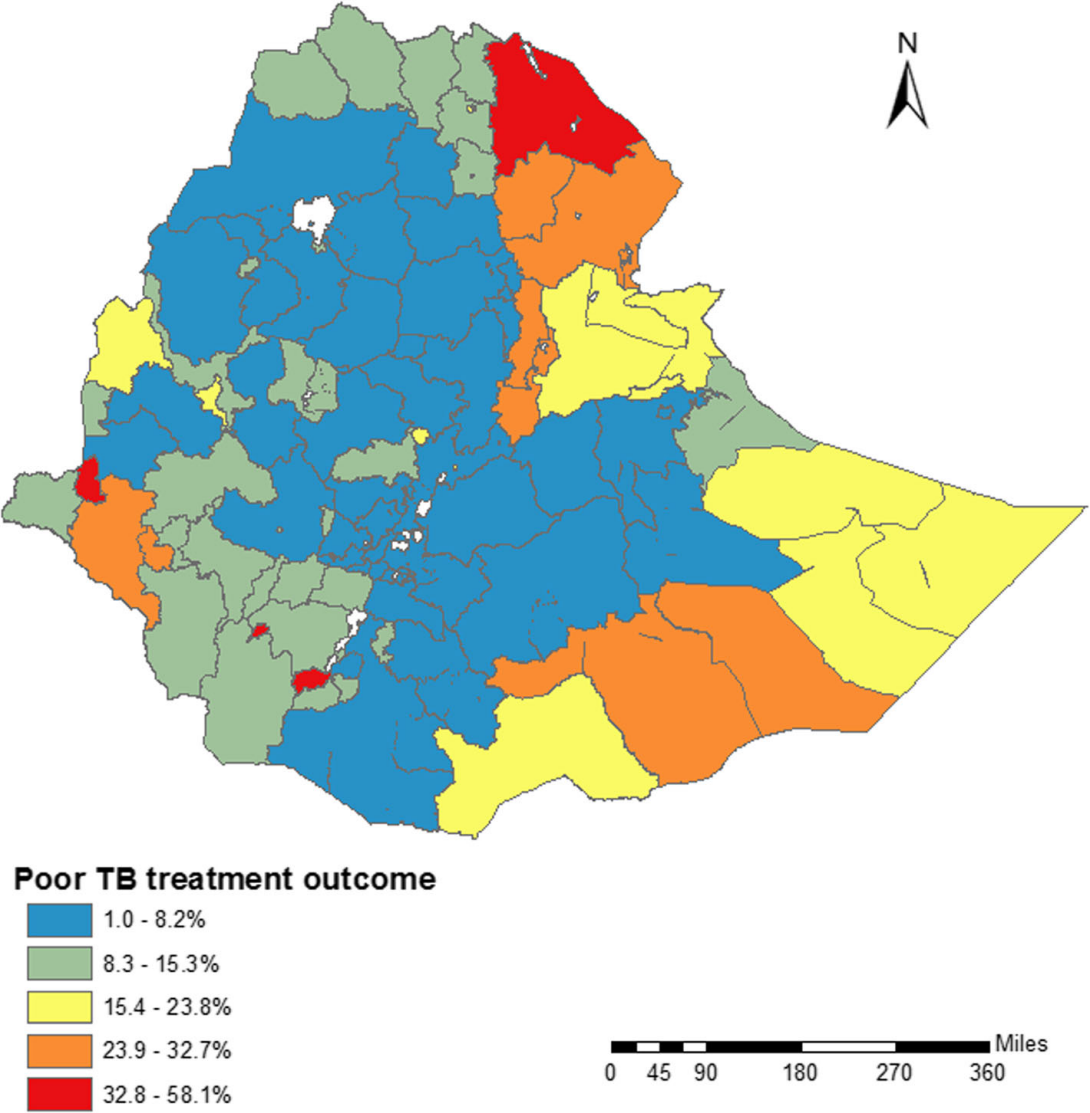
Choropleth map showing the geographical distribution of poor tuberculosis treatment outcome by zone in Ethiopia, June 2015 to June 2017

A total of 223,244 patients in 722 districts were reported to the national NTLCP (through the HMIS) during the period June 2015 and June 2017. Of these, 63,556 (28.5%) were cured, 139,633 (62.4%) completed treatment, 6716 (3.0%) died, 1459 (0.7%) had treatment failure, and 12,200 (5.5%) were lost to follow-up. The overall prevalence of poor TB treatment outcomes was 20,375 (9.0%). Figure 1 presents the spatial distribution of poor TB treatment outcomes in Ethiopia at the zone level. The prevalence of poor TB treatment outcomes varied from 1% in East Harerge Zone, Oromia region to 58% in Stang Special Zone, Gambelia region (Fig. 1). The districts with the highest prevalence of poor TB treatment outcomes were located in West and East Ethiopia in the Afar, Gambelia, and Somali regions, whereas districts with a lower prevalence of poor TB treatment outcomes were mainly located in the central part of Ethiopia including in the Oromia and Amhara regions.

### Spatial clustering of poor TB treatment outcomes in Ethiopia

**Fig. 2:**
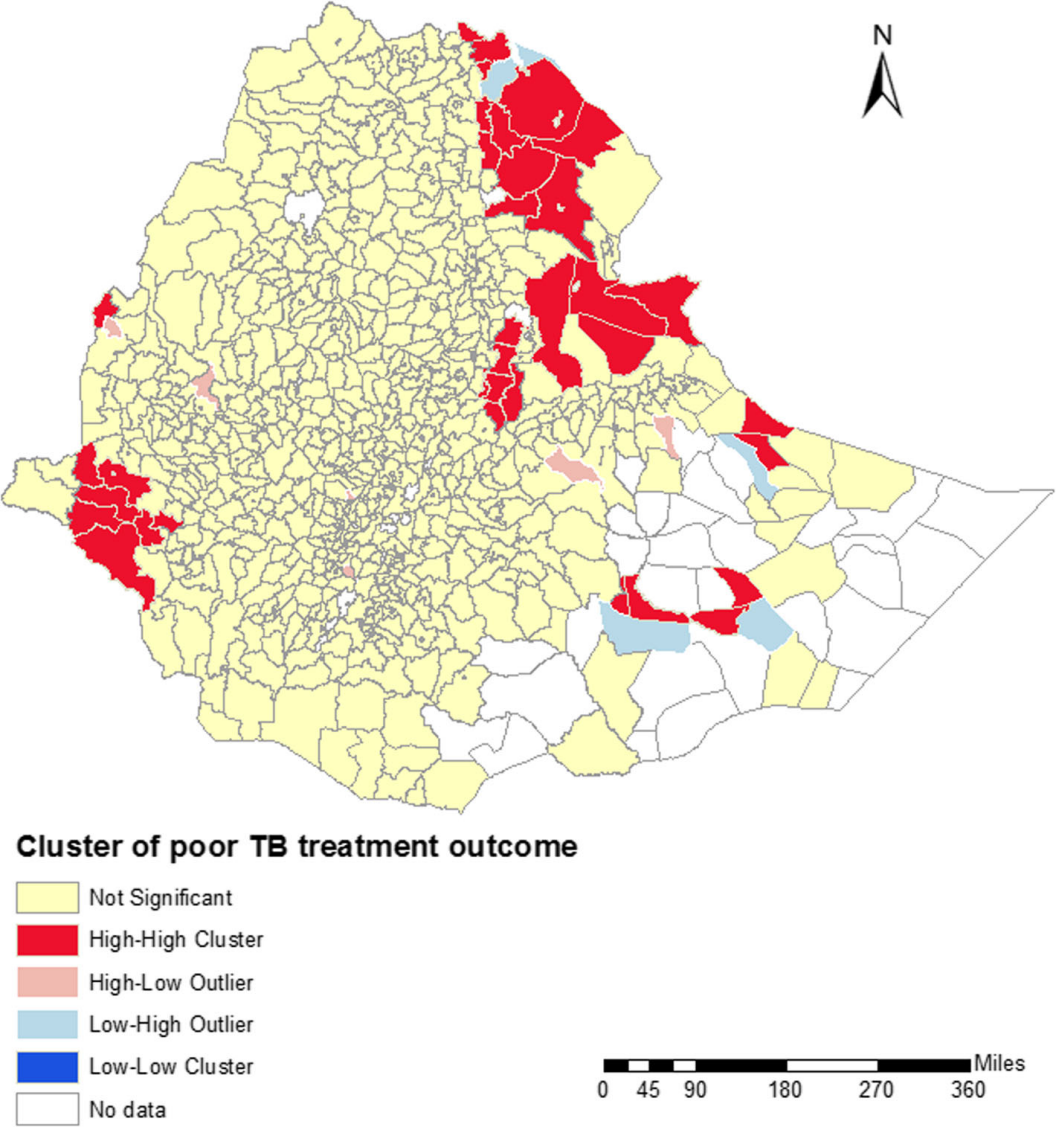
Spatial clustering of poor tuberculosis treatment outcomes at district-level in Ethiopia, 2015 to 2017, based on Local indicators of spatial association using Anselin Local Moran’s I statistic

**Fig. 3:**
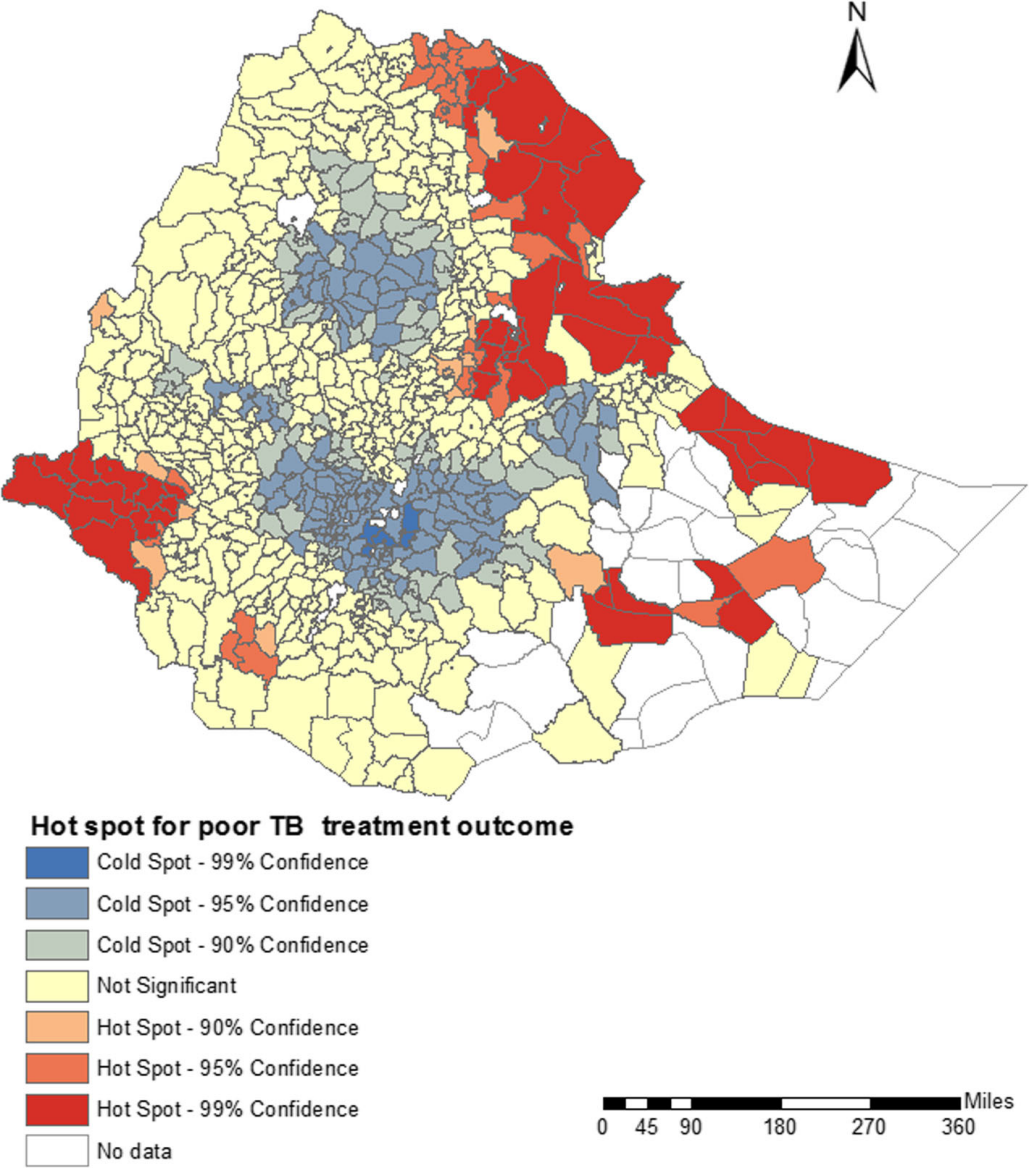
Spatial clustering of poor tuberculosis treatment outcomes at district-level in Ethiopia, 2015 to 2017, based on the Getis-Ord Gi* statistic

Spatial clustering of poor TB treatment outcomes was observed in Ethiopia both at district (Global Moran’s I = 0.06, *p*-value = < 0.001) and zone levels (Global Moran’s I = 0.10, *P*-value = 0.04). Figure 2 shows that high-high clusters of poor TB treatment outcomes were observed in districts located in Gambelia, Afar and Somalia regions. Similarly, Fig. 3 shows that hot spots of poor TB treatment outcomes were detected in districts located in Gambelia, Afar and Somalia regions. The districts where hot spots and high clustering of poor TB treatment outcomes were observed are located in the border regions of Ethiopia, and are characterized by high annual mean temperature, and low levels of health care access. Cold spots of poor TB treatment outcomes were detected in central Ethiopia, mainly in Oromia and Amhara regions (Fig. 3).

### Factors associated with poor TB treatment outcomes

Table 3 presents the univariate analysis showing the association between poor TB treatment outcomes and ecological-level socio-economic, behavioural, health care access and climatic variables. Table 4 shows the Bayesian spatial and non-spatial models for poor TB treatment outcomes with and without covariates. The model that contained covariates with an unstructured random effect was the best-fitting, most parsimonious model according to the value of the DIC statistic. The proportion of the population with low wealth index ((Odds Ratio (OR): 1.01; 95%CI: 1.0, 1.01), the proportion of the population with poor knowledge about TB (OR: 1.02; 95%CI: 1.01, 1.03), and higher annual mean temperature per degree Celsius (OR: 1.15; 95% CI: 1.08, 1.21) were positively associated with a poor TB treatment outcome. When the independent variables were incorporated into the model, spatial clustering was not apparent in the residuals (Global Moran’s *I* = − 0.01; *p*-value = 0.23), indicating that the covariates could explain the spatial clustering in poor TB treatment outcomes.

**Table 3:** Univariate model for poor tuberculosis treatment outcomes at the zone level in Ethiopia, 2015–2017

Independent variables	Coefficient (95% CI)	P-value
Socio-economic factors		
Low wealth index	0.01 (0.005, 0.016)	< 0.001
Population density	0.00002 (−0.00004, 0.00009)	0.429
Dependency ratio	−1.01 (−1.85, − 0.192)	0.016
Average number of persons per room	0.23 (0.11, 0.350)	0.001
Unemployed population	0.0535 (0.025, 0.082)	< 0.001
Adult literacy rate	−0.0088 (− 0.013, − 0.0002)	0.045
Behavioural factors		
Chat chewing	−0.011 (− 0.02, − 0.003)	0.011
Alcohol drinking	− 0.0039 (− 0.009, 0.001)	0.115
Health care access		
Health care access problem	−0.0001 (− 0.01, 0.008)	0.995
Poor knowledge towards TB	0.003 (0.0015, 0.009)	0.046
Poor attitude towards TB	0.002 (−0.007, 0.012)	0.653
Climatic factors		
Enhanced vegetation index	−0.0001 (− 0.0003, −6.59e-06)	0.041
Rainfall	−0.0001 (− 0.0003, −4.93e-06)	0.044
Aridity	− 0.0001 (− 0.0001, − 0.00003)	0.001
Mean temperature	0.133 (0.09, 0.168)	< 0.001

**Table 4:** Bayesian logistic regression model for the association of socio-economic, behavioural, climatic condition and spatially structured and unstructured random effect at the zone level with poor tuberculosis treatment outcomes in Ethiopia, 2015 to 2017

Variables	Unstructured model	Structured model	Structured & unstructured model	Model without covariates
	OR, Posterior Mean (95% CrI	OR, Posterior Mean (95% CrI	OR, Posterior Mean (95% CrI	
Chat chewing	0.99 (0.98, 1.00)	0.99 (0.98, 1.00)	0.99 (0.98, 1.00)	–
Low wealth index	**1.01 (1.00, 1.01)**	**1.01 (1.00, 1.01)**	**1.01 (1.00, 1.01)**	–
Knowledge towards TB	**1.02 (1.01, 1.03)**	**1.013 (1.00, 1.03)**	**1.02 (1.01, 1.03)**	–
Mean temperature	**1.15 (1.08, 1.21)**	**1.12 (1.045, 1.18)**	**1.14 (1.08, 1.20)**	–
Constant (alpha)	−5.57 (−6.64, −4.39)	−5.04 (− 6.17, −3.74)	− 5.48 (− 6.51, − 4.37)	−2.11 (−2.14, −2.07)
Heterogeneity				
Unstructured variance	0.63 (0.53, 0.74)	–	0.24 (0.028, 0.83)	
Structured variance	–	1.264 (1.07, 1.50)	0.59 (0.42, 0.73)	1.57 (1.34, 1.84)
DIC	746.0	764.5	752.0	760.8

*CrI* credible interval, *DIC* deviance information criterion, *OR* odds ratio

The boldface indicate statistically signficant variables

## Discussion

This nation-wide study identified substantial variability in the prevalence of poor TB treatment outcomes across districts and zones of Ethiopia. These inequalities were associated with underlying differences in multiple area-level socioeconomic and climatic factors and knowledge of TB. For example, low wealth index, high annual mean temperature, and poor knowledge about TB were associated with poor TB treatment outcomes. Moreover, significant geographic clustering was apparent, with poor TB treatment outcome hot spots in the border regions and cold spots in the central regions of Ethiopia. Notably, the selection of the non-spatial Bayesian model as the best-fitting model indicates that the spatial clustering of poor treatment outcomes observed in the exploratory analysis was largely explained by the model covariates. The associations between poor TB treatment outcome and markers of socioeconomic status, climatic factors, and health care access have important clinical and public health implications, suggesting high-risk areas that could be targets for enhanced activities for prevention and control of TB.

Geographic variability exists for multiple health conditions in Ethiopia, particularly infectious diseases [8, 28–30]. We previously demonstrated that rates of paediatric TB and multidrug-resistant TB vary across districts in Ethiopia [8, 9]. However, to our knowledge, no previous published study has assessed spatial variability in TB treatment outcomes in Ethiopia. In Iran, Kolifarhood et al. reported variability in successful TB treatment outcomes across two districts of Urmia [10]. A study in Argentina found spatial clustering of loss to follow up across districts with low socio-economic status and difficult access to public transportation [11]. We found that the overall prevalence of poor TB treatment outcomes was 9%, which is consistent with the global TB report [2, 21]. However, when we assessed poor TB treatment outcomes at the district and zone levels, these outcomes ranged from 1 to 58%. This finding highlights the high degree of variability in poor TB treatment outcomes that exists within just one country, masked by an overall national TB treatment success rate (84%) [16].

Hot spots of poor TB treatment outcomes were detected in districts located in Afar, Somalia and Gambelia regions. These regions are located in the border areas of Ethiopia and are characterised by high air temperatures. For example, the maximum temperature reaches 40 °C in the Somalia region and 48 °C in the Afar region. Our findings also showed that higher annual mean temperature is associated with poor TB treatment outcomes. Temperature may impact on the efficacy of the drugs that are used to treat TB, and therefore, transmission. However, this relationship could be also confounded by other factors. Clearly, future studies are needed to better understand why increased environmental air temperatures are associated with poor TB treatment outcomes and what interventions are best suited toward preventing poor TB treatment outcome in hot environments.

The majority of the people who live in Afar and Somalia regions, where hot spots of poor TB treatment outcomes were observed, are pastoralists who are dependent on livestock for their income. These group of people are nomads who move from place to place to get feed for their livestock [27]. Since many of the settlements and villages in these regions are of a temporary nature, the development of basic infrastructure (i.e. schools, health care facilities, and water supply) is challenging [27]. As a result, in these regions, infrastructure and health care services are underdeveloped [27]. Designing TB treatment programmes for nomadic communities, and for people living in border areas that take migratory practices into account, could be important to increase access to TB care and achieve successful TB treatment outcomes. A study conducted in Nigeria showed that active case finding for TB among nomadic populations increased case notification and successful treatment outcomes [31]. This approach is particularly important for Ethiopia to achieve the END TB targets of ending TB related deaths, transmission and catastrophic costs by 2035 [32].

Variability in the prevalence of poor TB treatment outcomes was significantly associated with underlying variability in area-level socioeconomic status. This finding is consistent with previous studies assessing such relationships with TB treatment outcomes in Ethiopia [33, 34] Such findings are not surprising given the extensive literature on the influence of individual-level socioeconomic deprivation on TB prevalence [35–37].

The observed associations between area-level socioeconomic status and area-level prevalence of poor TB treatment outcome highlight the potential benefit of identifying hotspot targets for clinical and public health interventions. For example, provision of health education for targeted communities is important to increase the TB-related knowledge, a consideration supported by our findings [38]. Up to date information on district or zone-level TB “hot spots” could be used to inform healthcare planning, including prioritisation of resources that might strengthen the DOTS program.

This study has a number of limitations. First, the prevalence of poor TB treatment outcomes for a district or zone may vary with time. We used the most recent 2 years of data reported to the Ministry of Health after the full implementation of the HMIS. Second, although the data covered all zones and regions of Ethiopia, there were no TB data available for 32 districts. Third, the ecological study design did not allow us to determine causation or directionality of association between poor TB treatment outcomes and zone and district–level characteristics. Fourth, we depended on data reported to the health care facilities. However, all patients with TB may not visit the health care facilities and thus we were unable to directly measure the prevalence of poor TB treatment outcomes in the community, including longer-term follow-up of patients who “completed treatment” to determine long-term outcomes. Fifth, since the study was based on district-level aggregated data, some important demographic and clinical variables such as age, sex, weight, human immunodeficiency virus (HIV) status, initial bacterial load, and drug susceptibility patterns were not available in the reports and therefore were not included in our study. Demographic variables such sex and age may have a significant effect on the geographical distribution of poor TB treatment outcomes; however, such data were not available and thus not included in the study. Sixth, the assessment of access to health care was based on data obtained only from women, which might have introduced bias.

## Conclusions

The prevalence of poor TB treatment outcomes varied substantially across districts and zones in Ethiopia. This variability was significantly associated with underlying differences in zone–level socioeconomic status, knowledge about TB, and mean annual temperature. Hot spots of poor TB treatment outcomes were observed in districts located in the border region of Ethiopia (i.e. Afar, Somalia, and Gambelia regions), where pastoralist communities live. Cold spots were present in districts located in central Ethiopia (i.e. in Amhara and Oromia region). Clinical and public health interventions should be targeted to hot spot areas to improve TB treatment outcomes and to achieve the national End-TB Strategy targets.

## Additional file

Additional file 1: **Table S1.** The spatial models constructed using WinBUGS software, version 1.4. (DOCX 25 kb)

## Abbreviations

CAR: Conditional autoregressive
DIC: Deviance information criterion
DOTS: Directly observed therapy, short-course
EDHS: Ethiopian Demographic and Health Survey
GBD: Global Burden of Disease
HIV: Human immunodeficiency virus
HMIS: Health management information system
LISA: Local indicators of spatial association
MCMC: Markov chain Monte Carlo
MDG: Millennium Development Goal
NHMRC: National Health and Medical Research Council
NTLCP: National TB and leprosy control program
OR: Odds ratio
TB: Tuberculosis
VIF: Variance inflation factors
WHO: World Health Organization
