# Supplementary material for: Mapping tuberculosis treatment outcomes in Ethiopia
Source: BMC Infect Dis. 2019 May 28;19:474. doi: 10.1186/s12879-019-4099-8 (PMC6540408; doi:10.1186/s12879-019-4099-8)
Supplement: Supplementary file 1 — Table S1. The spatial models constructed using WinBUGS software, version 1.4. (DOCX 25 kb) [file 12879_2019_4099_MOESM1_ESM.docx]

**Supplemental files**

**Table S1:** The spatial models constructed using WinBUGS software, version 1.4

| Unstructured model | Spatially structured model | Spatially structured & unstructured model | Spatially structured model without covariate |
| --- | --- | --- | --- |
| model {  for (i in 1:88) {  r[i] ~ dbin(p[i],n[i]) logit(p[i]) <- alpha + beta1*Chat[i] + beta2*Low_wealth[i] + beta3* TB_know[i] + beta4*Mean_Temp[i] + u[zon[i]]  u[i]~dnorm(0.0, tau.u)  } | model {  for (i in 1:88) {  r[i] ~ dbin(p[i],n[i])  logit(p[i]) <- alpha + beta1*Chat[i] + beta2*Low_wealth[i] + beta3*TB_know[i] + beta4*Mean_Temp[i] + v[zon[i]] }  v[1:88] ~ car.normal(adj[], weights[], num[], tau.v); for (j in 1:442){weights[j] <- 1 } | model {  for (i in 1:88) {  r[i] ~ dbin(p[i],n[i])  logit(p[i]) <- alpha + beta1*Chat[i] + beta2*Low_wealth[i] + beta3*TB_Know[i] + beta4*Mean_Temp[i] + u[zon[i]] + v[zon[i]]  }  for (i in 1: 88) {  u[i]~ dnorm(0.0, tau.u)  }  v[1:88] ~ car.normal(adj[], weights[], num[], tau.v);  for (j in 1:442){weights[j] <- 1  } | model {  for (i in 1:88) {  r[i] ~ dbin(p[i],n[i])  logit(p[i]) <- alpha + v[zon[i]]  }  v[1:88] ~ car.normal(adj[], weights[], num[], tau.v);  for (j in 1:442){weights[j] <- 1 } |
| alpha ~ dflat()  beta1 ~ dnorm(0,0.000001)  beta2 ~ dnorm(0,0.000001)  beta3 ~ dnorm(0,0.000001)  beta4 ~ dnorm(0,0.000001)  tau.u~ dgamma(0.001, 0.001)  tau.v~ dgamma(0.001, 0.001)  OR1 <- exp(beta1)  OR2 <- exp(beta2)  OR3 <- exp(beta3)  OR4 <- exp(beta4)  sigma.u <- sqrt(1/tau.u)  sigma.v <- sqrt(1/tau.v) }  **#Initial values**  list( alpha = 0, beta1 = 0, beta2 = 0, beta3 = 0, beta4 = 0, tau.u = 0.5, tau.v = 0.5) | | | |
